# Independent Tissue-Based Biomarkers in Endometrioid Endometrial Cancer: Tumor Budding in Microsatellite Instability and WHO Grading in Copy-Number-Low Patients

**DOI:** 10.3390/cancers15153832

**Published:** 2023-07-28

**Authors:** Fabian Stögbauer, Barbara Geß, Christine Brambs, Manuela Lautizi, Tim Kacprowski, Iordanis Ourailidis, Holger Bronger, Marion Kiechle, Aurelia Noske, Gisela Keller, Moritz Jesinghaus, Christopher Poremba, Wilko Weichert, Melanie Boxberg

**Affiliations:** 1 Institute of Pathology, School of Medicine, Technical University of Munich (TUM), 81675 Munich, Germany; fabian.stoegbauer@tum.de (F.S.); manuela.lautizi@tum.de (M.L.); aurelia.noske@tum.de (A.N.); gisela.keller@tum.de (G.K.); wilko.weichert@tum.de (W.W.); 2 Department of Obstetrics and Gynecology, Technical University of Munich, 81675 Munich, Germany; barbara.gess@mri.tum.de (B.G.); holger.bronger@tum.de (H.B.);; 3 Lucerne Cantonal Hospital, Department of Obstetrics & Gynecology, Division of Gynecologic Oncology, 6000 Lucerne, Switzerland; christine.brambs@luks.ch; 4 Chair of Experimental Bioinformatics, TUM School of Life Sciences Weihenstephan, Technical University of Munich, 85354 Freising, Germany; 5 Division Data Science in Biomedicine, Peter L. Reichertz Institute for Medical Informatics, TU Braunschweig and Hannover Medical School, 38106 Brunswick, Germany; t.kacprowski@tu-braunschweig.de; 6 Braunschweig Integrated Centre of Systems Biology (BRICS), 38106 Brunswick, Germany; 7 Institute of Pathology, Heidelberg University Hospital, 69120 Heidelberg, Germany; iordanis.ourailidis@med.uni-heidelberg.de; 8 German Cancer Consortium (DKTK) Partner Site Munich, 81675 Munich, Germany; 9 Institute of Pathology, University Hospital Marburg, Baldingerstraße, 35043 Marburg, Germany; moritz.jesinghaus@uni-marburg.de; 10 Pathologie München-Nord, 80992 Munich, Germany; poremba@pathologie-muenchen.de

**Keywords:** endometrial cancer, molecular subtypes, MSI, CN-low, tissue based biomarkers, tumor budding, prognosis, lymph node metastasis

## Abstract

**Simple Summary:**

Rrisk assessment of microsatellite instability (MSI) and copy-number (CN)-low endometrial adenocarcinomas constitutes a major challenge. We aimed to identify tissue-based morphologic biomarkers that might help in the prognostic stratification, comprehensively analyzing one finding cohort (TCGA-UCEC) and two independent validation cohorts. Histomorphologic parameters (WHO grading, tumor budding (TB), tumor–stroma ratio, tumor-infiltrating lymphocytes (TIL), “microcystic, elongated, fragmented” (MELF) pattern) were analyzed. For each quantitative parameter, a two-tiered system was developed utilizing systematically determined cutoffs. In MSI tumors, TB (≥3 buds/high-power field) was detected to be an independent prognostic factor for inferior outcomes and lymph node metastases. The finding was confirmed in two validation cohorts. For CN-low tumors, solely WHO grading was independently prognostic with inferior outcomes for high-grade tumors. Therefore, we propose the utilization of TB and WHO-based grading, two tissue-based and easy-to-assess biomarkers, in MSI/CN-low endometrial carcinomas for improved clinical management.

**Abstract:**

The molecular characterization of endometrial endometrioid adenocarcinomas has provided major advances in its prognostic stratification. However, risk assessment of microsatellite instability (MSI) and copy-number (CN)-low cases remains a challenge. Thus, we aimed to identify tissue-based morphologic biomarkers that might help in the prognostic stratification of these cases. Histomorphologic parameters (WHO grading, tumor budding (TB), tumor–stroma ratio (as a quantitative description of stromal desmoplasia), tumor-infiltrating lymphocytes (TIL), “microcystic, elongated, fragmented” (MELF) pattern) were analyzed in resection specimens of the TCGA-UCEC cohort (*n* = 228). For each quantitative parameter, a two-tiered system was developed utilizing systematically determined cutoffs. Associations with survival outcomes were calculated in univariate and multivariate analysis and validated in two independent cohorts. In MSI tumors, only TB remained an independent prognostic factor. TB (≥3 buds/high-power field) was associated with inferior outcomes and with lymph node metastases. The prognostic significance of TB was confirmed in two validation cohorts. For CN-low tumors, established grading defined by the WHO was independently prognostic with inferior outcomes for high-grade tumors. The evaluation of TB might help in identifying MSI-patients with unfavorable prognosis who, e.g., could benefit from lymphadenectomy. WHO-based grading facilitates independent prognostic stratification of CN-low endometrioid adenocarcinomas. Therefore, we propose the utilization of TB and WHO-based grading, two tissue-based and easy-to-assess biomarkers, in MSI/CN-low endometrial carcinomas for improved clinical management.

## 1. Background

Endometrial cancer is the sixth most prevalent malignancy in women, with approximately 382,000 new cases annually worldwide [1]. Endometrioid adenocarcinoma is the main histological type of endometrial carcinoma [2]. In an effort to subclassify endometrial cancer, The Cancer Genome Atlas (TCGA) Program identified four distinct molecular subtypes of endometrial cancer: Polymerase ε (*POLE*) ultramutated, microsatellite instable (MSI) hypermutated, copy-number-low (CN-low) and copy-number-high (CN-high) cases [3]. Based on this approach, Talhouk et al. proposed an algorithm applicable for routine pathology utilizing the immunohistochemical status of mismatch repair proteins and p53 expression combined with *POLE* mutational status as surrogate for the otherwise elaborate and expensive genomic characterization [4]. Regarding survival prediction, it could be shown that *POLE* mutated endometrial carcinomas show superior survival rates, while p53 abnormal cases (equivalent to CN-high) are associated with poor outcomes [5]. In contrast, mismatch-repair-deficient (equivalent to MSI) and CN-low cases (*POLE* wildtype; p53 wildtype; mismatch-repair-proficient), the two largest subgroups, are associated with intermediate prognostic outcomes, and prognostic stratification of patients is a major challenge [6,7]. Currently, risk assessment, and accordingly, treatment planning (e.g., administration of radiotherapy or chemotherapy) of patients with endometrioid adenocarcinomas are mainly based on the tumor stage, grade and lymphovascular space invasion (LVSI) [8,9,10]. However, additional reliable biomarkers that would allow for further prognostic stratification of patients with MSI and CN-low endometrioid carcinomas are currently not established in daily clinical practice [11,12]. Moreover, due to the high morbidity associated with lymphadenectomy, the identification of patients who might profit from lymphadenectomy is challenging [13]. Promising potential histomorphologic parameters, which have shown their prognostic significance in various studies on other solid tumor entities, comprise tumor budding (TB), stroma content/stromal desmoplasia and immune cell infiltration in terms of tumor infiltrating lymphocytes (TIL) [14,15,16]. The prognostic relevance of TB could be shown, e.g., in colorectal cancer, lung cancer and head and neck cancer [17]. For endometrial cancer, so far, only a few studies have evaluated TB (reviewed in [18]); however, those studies differed regarding the cutoffs applied for scoring TB [19,20]. Furthermore, while Qi et al. analyzed a grading scheme composed of TB and the “microcystic, elongated, fragmented” (MELF) pattern and obtained the worst outcomes for patients whose tumors show both TB and MELF; analysis was conducted irrespective of TCGA-defined molecular subgroups [20]. The same issue is a limitation of a recent study by Okcu et al. [21].

Similarly, high amounts of tumor stroma and low amounts of TIL were correlated with inferior outcomes in tumors of the gastrointestinal tract, the oral cavity and melanomas [22,23,24].

Taken together, data on the applicability of the above-mentioned histomorphologic parameters in endometrioid adenocarcinomas and especially in MSI and CN-low cases are scarce [19,25,26].

Aiming to obtain comprehensive data concerning the above-mentioned biomarkers in the context of TCGA-defined molecular subgroups, we assessed these histomorphologic parameters in a large cohort of patients with MSI and CN-low endometrioid adenocarcinomas and evaluated their prognostic significance. Subsequently, the prognostic relevance of potential biomarkers was validated in two independent cohorts.

The overarching aim of our study was to identify potential biomarkers that might pave the way to a better prognostic stratification of patients, improving treatment planning and follow-up procedures in clinical practice.

## 2. Methods

### 2.1. Study Cohort

Uterine endometrioid adenocarcinomas of the TCGA program (“TCGA-UCEC”) were utilized [3]. Diagnostic H&E stained slides were downloaded from the GDC data portal (https://portal.gdc.cancer.gov (accessed on 9 January 2022)) using the GDC Data Transfer Tool (v1.6.1) on 9 January 2022 [27]. Clinical data were downloaded from “cBioPortal for Cancer Genomics” (https://www.cbioportal.org (accessed on 9 January 2022)) on 19 July 2021 and updated on 6 June 2022. Subsequently, cases with insufficient tumor cell content or lack of adjacent stroma, a different tumor entity and inferior scan quality were excluded. After reviewing molecular subtyping, all available MSI and CN-low tumors were included. The final study cohort consisted of 228 patients with primary endometrioid endometrial adenocarcinoma with neither a history of prior malignancy nor neoadjuvant treatment. Detailed clinicopathologic characteristics of the study cohort are shown in Table 1. Disease-specific survival (DSS), overall survival (OS) and progression-free survival (PFS) data were available for 227 cases (99.6%). Median survival times were 32.1 months, 32.1 months and 3.7 months, respectively.

### 2.2. Validation Cohorts

To validate the prognostic significance of TB in MSI patients, we utilized our clinically well-characterized independent cohort of endometrioid adenocarcinomas (treated with hysterectomy) that already has been published [28]. From the original MSI cohort (*n* = 47) three cases had to be excluded because TB could not be evaluated due to small amounts of tumor or insufficient adjacent stroma. This resulted in a final cohort consisting of 44 cases with primary endometrial endometrioid adenocarcinomas (MSI; without a history of prior malignancy or neoadjuvant treatment, Table 1). Data regarding OS and PFS were available for all 44 cases (100%) with 41.5 months median survival time for DSS, OS and PFS.

For further validation of the findings, a second independent cohort of patients with endometrioid adenocarcinomas of the Clinical Proteomic Tumor Analysis Consortium (CPTAC) program was utilized [29]. Here, clinical data were downloaded from the GDC Data Portal using R-library “TCGAbiolinks” on 19 July 2021, and H&E stained slides were downloaded from The Cancer Imaging Archive (https://www.cancerimagingarchive.net/ (accessed on 9 January 2022)) [30,31,32,33]. Classification into subtypes (“POLE”, “CNV-high”, “CNV-low” and “MSI”) was conducted in accordance with Dou et al. [29], and only cases that were classified as MSI (*n* = 25) were included in the validation cohort. Clinicopathologic data of the CPTAC validation cohort are depicted in Table 1. Median survival times for OS and DSS were 24.4 months each.

### 2.3. Histomorphologic Parameters Analyzed

Histomorphologic grading was conducted in line with the recommendations of the current edition of the WHO Classification of Tumours—Female Genital Tumours [34]. Cases were subtyped into low-grade (G1, G2) vs. high-grade (G3) carcinomas depending on percentage of glandular formation and nuclear grade.

TB was defined as the detachment of four or fewer tumor cells from the main tumor mass infiltrating into the adjacent stroma [19]. For the assessment of TB, the whole slide was scanned at low-power magnification to determine the area with highest budding activity. Subsequently, the number of budding foci in the hotspot area in 40× magnification in 1 high-power field (HPF) and in 10 consecutive HPFs starting in the hotspot area were recorded. Both budding foci in the tumor center and at the invasive front were considered for the assessment of TB.

Minimal cell nest size (MCNS) was defined as the minimal number of cohesive tumor cells detached from the main tumor taking into account the whole tumor region. Applying this definition, MCNS of 1 up to clusters of 4 tumor cells represent buds (see above), with one single infiltrating cell termed as single cell invasion.

The tumor–stroma ratio (TSR) was determined as previously described [35]. It represents the proportion of tumor stroma compared to the proportion of carcinoma infiltrate within the area of highest stromal content, and is a quantitative measure of stromal desmoplasia. The term “stromal desmoplasia” exactly describes the morphologic pattern for which Kemi et al. introduced the term TSR in gastric cancer and Panayiotou et al. [25] applied it in the context of endometrial cancer. For reasons of comparability with previous studies, the abbreviation “TSR” as a measure of stromal desmoplasia is hereafter applied.

For evaluation of stromal desmoplasia/TSR, the invasive front of the tumor was analyzed at 20× magnification and the area with the highest stroma content was chosen. Here, invasive tumor cells had to be visible at all four edges of the field. Subsequently, the area of invasive tumor and of stroma was assessed and the ratio between both parameters was recorded. Stromal desmoplasia (TSR) could only be assessed in cases where the invasive edge was located on the digitized slide (*n* = 224).

For the determination of inflammation in terms of tumor-infiltrating lymphocytes (TIL) a slightly modified version of the algorithm proposed by Hendry et al. was utilized [36]. In short, the whole area within the borders of the tumor on a slide was analyzed. Subsequently, the ratio between the area covered by TIL and the area covered by tumor cells was calculated.

Areas of necrosis were excluded from the determination of TSR and TIL.

The percentage of gland formation was determined as the proportion of tumor cells with a glandular pattern of growth among all tumor cells on a slide (excluding areas with squamous metaplasia).

MELF pattern of invasion was defined as myoinvasive tumor glands with a microcystic or slit-like appearance and flattened epithelial lining or tumor cells located in edematous or myxoid stroma and evaluated, as defined, at the invasive front [37]. The MELF pattern could therefore only be determined in cases where the invasive front was included on the slides (*n* = 224).

Lymphovascular invasion was recorded in case of intraluminal tumor manifestations in lymphatic vessels [38].

**Figure 1:**
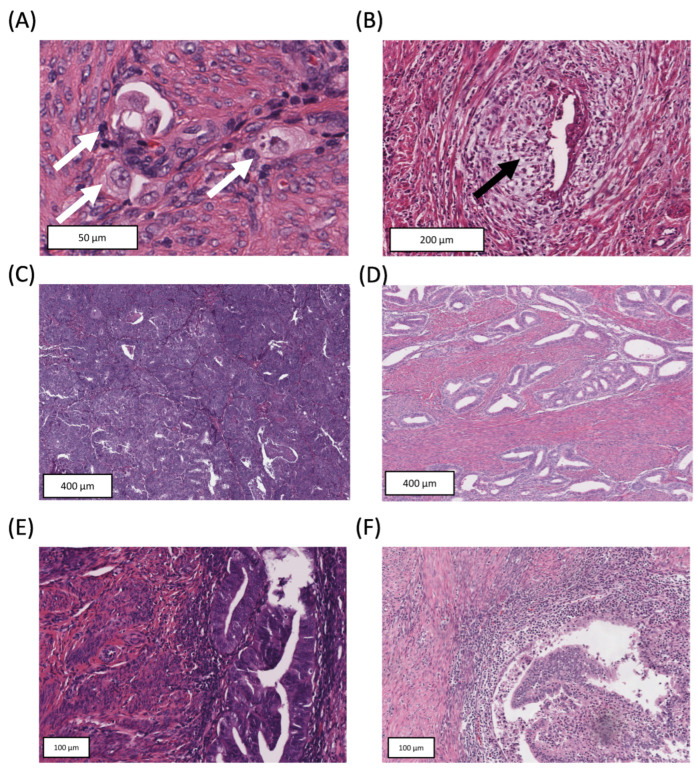
H&E stained slides of histomorphologic parameters. Tumor with budding activity (white arrows) (**A**) and tumor with MELF pattern of invasion (black arrow) (**B**). Tumor with high stromal desmoplasia (tumor–stroma ratio) (**C**) and tumor with low stromal desmoplasia (tumor–stroma ratio) (**D**). Tumor with low-grade tumor-infiltrating lymphocytic infiltrate (TIL) (**E**) and tumor with high-grade TIL (**F**).

Exemplary illustrations of the histomorphologic parameters are shown in Figure 1.

All available slides per patient were evaluated for the histomorphologic parameters. All analyses were conducted on digitized H&E stained slides on a standard monitor (Fujitsu B24T-7, Fujitsu Limited, Tokyo, Japan, resolution 1920 × 1080) by two experienced pathologists blinded to clinicopathologic data (FS, MB). Discrepant cases were classified based on an agreement between both raters. For the evaluation of digitized slides Aperio ImageScope (version 12.4.0.7018; Leica Biosystems Nussloch GmbH, Nussloch, Germany) and QuPath (version 0.3.0) were utilized [38,39]. One digital HPF comprised an area of 116,160 µm^2^, which corresponds to a field diameter of 0.38 mm in light microscopy. 

### 2.4. Cutoff Determination

For histomorphologic parameters (TB in 1 and 10 HPF, MCNS, TSR/stromal desmoplasia and TIL) the prognostically most significant cutoff values were determined. Therefore, a freely available online tool (“Cutoff Finder” [40]) was utilized to dichotomize patients, leading to optimal prognostic significance regarding disease-specific survival.

### 2.5. ITBCC Grading Scheme

To compare the above defined 2-tier grading scheme for TB with the 3-tier grading scheme recommended by the International Tumor Budding Consensus Conference (ITBCC) for colorectal cancer, we stratified the patients of the TCGA study cohort according to the guidelines described by the ITBCC [41]. Therefore, scores for TB in an evaluated 20× field were multiplied with the normalization factor reported by the ITBCC to assure the comparability of results obtained from different field areas of microscopy [41].

### 2.6. Statistical Methods

Fisher’s exact test was applied for the comparison of nominally scaled and ordinally scaled data. The Mann–Whitney U test was used to test for differences in continuously scaled data. Spearman’s ρ was determined to test for correlations between continuous variables. Survival analyses were conducted with the Kaplan–Meier method and comparisons of groups were calculated with the log-rank test. The influence of variables on survival was determined in univariate and multivariate analyses applying a Cox proportional hazard regression model. All statistical tests were conducted two-sided and *p*-values < 0.05 were regarded as significant. Statistical analyses were conducted with R (version 4.1.0) [42].

### 2.7. Ethics

The study was conducted in accordance with the Declaration of Helsinki. Ethics and policies regarding the TCGA cohort were originally published by the National Cancer Institute (https://www.cancer.gov/about-nci/organization/ccg/research/structural-genomics/tcga/history/policies (accessed on 9 January 2022)).

For the Munich validation cohort, the analyses were approved by the institutional review board (331/17).

Informed consent was obtained from all subjects involved in the study.

## 3. Results

### 3.1. Metrics of Histomorphologic Parameters

Median values determined in the TCGA cohort were 0.0 for TB (1 HPF and 10 HPF), 8.0 for MCNS, 3.5 for TSR and 8.0 for TIL. Corresponding interquartile ranges were 1.0 for TB (1 HPF and 10 HPF), 13.3 for MCNS, 3.3 for TSR and 11.0 for TIL. Medians, interquartile ranges, means, standard deviations, minimums and maximums are summarized in Supplementary Table S1.

Subsequently, patients were stratified according to the results of the histomorphologic analysis utilizing “Cutoff Finder” [40]. This resulted in a two-tier classification scheme for each histomorphologic parameter. The cutoffs identified by “Cutoff Finder” and the resulting composition of subgroups are shown in Supplementary Table S2. 

### 3.2. Mutual Correlations of Histomorphologic Parameters

Spearman’s ρ was calculated to detect mutual correlations between histomorphologic parameters (Supplementary Figure S1). TB (1 HPF and 10 HPF) was negatively correlated with MCNS (ρ = −0.84, *p* < 0.001 and ρ = −0.81, *p* < 0.001) and the percentage of gland formation (ρ = −0.40, *p* < 0.001 and ρ = −0.44, *p* < 0.001). It was positively correlated with lymph node metastases (ρ = 0.22, *p* < 0.001 and ρ = 0.11, *p* < 0.05). Furthermore, TB in 10 HPF was positively correlated with the depth of myometrial invasion (ρ = 0.15, *p* = 0.01) and with the number of buds in 1 HPF (ρ = 0.97, *p* < 0.001). TSR was negatively correlated with myometrial invasion in mm (ρ = −0.26, *p* = 0.04) and with TIL (ρ = −0.17, *p* < 0.001) and TIL infiltrate was negatively correlated with myometrial invasion in mm (ρ = −0.14, *p* = 0.02). Additionally, myometrial invasion was positively correlated with lymph node metastases (ρ = 0.27, *p* < 0.001) and negatively correlated with the percentage of gland formation (ρ = −0.32, *p* < 0.001). The percentage of gland formation was negatively correlated with lymph node metastases (ρ = −0.21, *p* = 0.01) and positively correlated with MCNS (ρ = 0.35, *p* < 0.001).

Additionally, mutual correlations between each histomorphologic parameter were calculated with Fisher’s exact test (Supplementary Table S3). TB in 1 HPF was associated with TB in 10 HPF, with MCNS and with the ITBCC grading scheme (*p* < 0.001, each). TB in 10 HPF was associated with MCNS (*p* < 0.001), LVSI (*p* = 0.006) and the ITBCC grading scheme (*p* < 0.001). The ITBCC grading scheme was associated with MCNS (*p* < 0.001), MELF (*p* = 0.004) and LVSI (*p* = 0.008).

### 3.3. Clinicopathologic Correlations of Histomorphologic Parameters

After stratification of patients concerning the results of the histomorphologic parameters associations with clinicopathologic data were calculated (Table 2).

Here, high-grade tumors showed more often an invasion of the outer myometrial half (*p* < 0.001), were more often lymph node-positive (*p* = 0.001), showed higher FIGO stages (*p* = 0.024), showed more often LVSI (*p* < 0.001), were more often incompletely resected (*p* < 0.001) and were more often MSI (*p* < 0.001). Similarly, high TB (1 HPF) was associated with positive lymph nodes (*p* < 0.001), higher FIGO stages (*p* = 0.026), higher tumor grade (*p* < 0.001), R1/R2 resection status (*p* < 0.001) and MSI (*p* = 0.036). High TB (10 HPF) was associated with higher pT stages (*p* = 0.045), positive lymph nodes (*p* = 0.007), higher tumor grade (*p* < 0.001), LVSI (*p* = 0.006), R1/R2 resection status (*p* = 0.002) and MSI (*p* = 0.007). A small MCNS was associated with lymph node metastases (*p* = 0.003), higher tumor grade (*p* < 0.001), a higher rate of R1/R2 resections (*p* = 0.002) and with MSI (*p* = 0.004). A low TSR was associated with higher pT (*p* = 0.012) and FIGO stages (*p* = 0.028), whereas high TIL was associated with MSI (*p* = 0.049).

### 3.4. Prognostic Significance of Histomorphologic Parameters in Univariate Survival Analysis

After stratification of patients using the two-tier system for every histomorphologic variable, log-rank tests were calculated for DSS, OS and PFS (Table 3). Calculated *p*-values of log-rank tests as well as median survival times are shown in Supplementary Table S4 and Supplementary Table S5, respectively.

**Figure 2:**
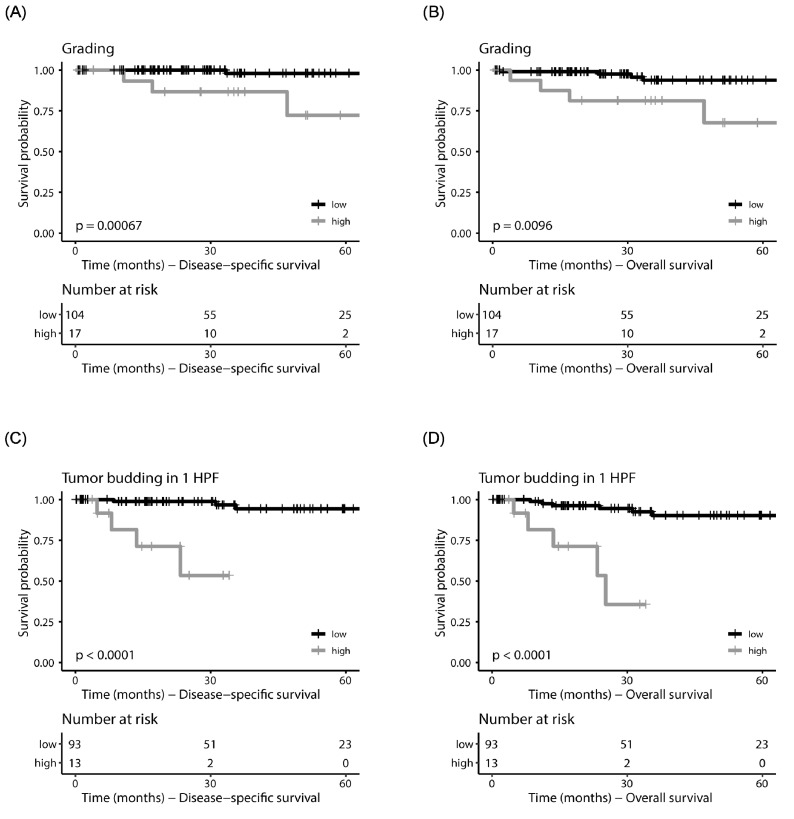
Kaplan–Meier plots for grading of the CN-low cases of the TCGA cohort. Disease-specific survival is shown in (**A**) and overall survival in (**B**). Kaplan–Meier plots for tumor budding in 1 HPF for the MSI cases of the TCGA cohort. Disease-specific survival is shown in (**C**), overall survival in (**D**).

WHO grading showed prognostic significance for DSS, OS and PFS in the whole TCGA cohort, for DSS and PFS in the MSI subgroup and for DSS and OS in the CN-low subgroup (Figure 2A,B).

TB (1 HPF/10 HPF) was prognostically significant in the whole TCGA cohort (DSS, OS and PFS) and in the MSI subgroup (DSS, OS and PFS; Figure 2C,D).

MCNS demonstrated prognostic significance for DSS and OS in the whole TCGA cohort (DSS and OS) and in the MSI subgroup (DSS and OS), whereas TSR demonstrated prognostic significance for DSS and OS in the whole TCGA cohort and in the MSI subgroup.

Furthermore, prognostically significant results could be obtained for TIL for DSS, OS and PFS in the CN-low subgroup and for MELF (DSS) in the whole cohort and in the MSI subgroup.

LVSI (DSS and PFS) was prognostically significant in the whole TCGA cohort, in the MSI subgroup and in the CN-low subgroup.

Patient stratification according to the ITBCC grading scheme showed prognostic significance for DSS, OS and PFS in the whole TCGA cohort and for DSS and OS in the MSI subgroup.

### 3.5. Prognostic Significance of Histomorphologic Parameters in Cox Proportional Hazard Analyses

In univariate analysis for DSS (Supplementary Figure S2) higher FIGO stages (HR: 6.80, *p* < 0.002), a higher grade (HR: 14.85, *p* < 0.001), presence of LVSI (HR: 6.50, *p* = 0.002), high TB 1/10 HPF (HR: 14.99/12.64, *p* < 0.001 each), a small MCNS (HR: 7.11, *p* = 0.001), low TSR (HR: 5.16, *p* = 0.040), presence of MELF (HR: 3.92, *p* = 0.042) and high budding according to the ITBCC scheme (HR: 9.97, *p* < 0.001) were associated with poor outcomes.

All parameters that showed significant results in univariate analysis (TB in 1 HPF, TB in 10 HPF, MCNS, TSR, MELF, ITBCC scheme) were included separately in multivariate analysis for DSS (besides age, FIGO stage, grading and LVSI).

In multivariate analysis of the whole cohort and of the CN-low subgroup, none of the histomorphologic parameters (TB in 1 and 10 HPF, MCNS, TSR, MELF, ITBCC scheme) remained independently prognostic. However, the only morphologic biomarker with independent prognostic relevance for the whole cohort as well as for the subgroup of CN-low tumors was WHO grading (when adjusting for age, FIGO stage, grading, LVSI and separately one of the histomorphologic parameters, Table 4).

**Figure 3:**
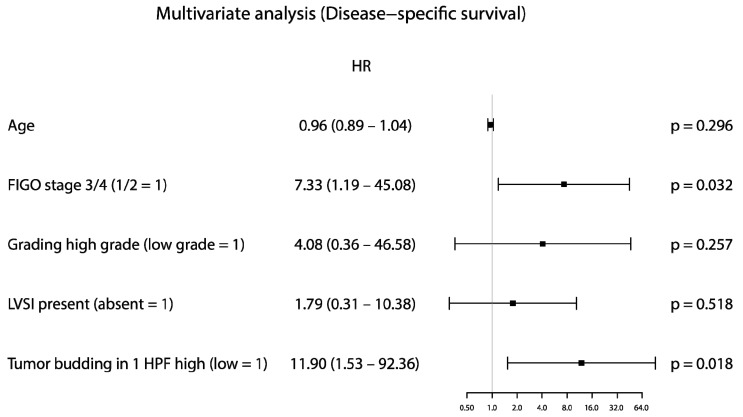
Forestplot for tumor budding in 1 HPF for the MSI subgroup of the TCGA cohort (disease-specific survival).

By contrast, in the subgroup of MSI tumors TB in 1 HPF (HR: 11.90, *p* = 0.018, Figure 3) and MELF pattern of invasion (HR: 14.14, *p* = 0.020) was proved to be an independent prognostic factor.

Further results of multivariate analysis for morphologic patterns with prognostic impact in univariate analysis are shown in Table 4.

As Qi. et al. [20] previously proposed a grading system including MELF and TB, we included these parameters (TB and MELF) in a subsequent multivariate analysis to probe their independent impact in a Cox regression model. Here, only TB was shown to be an independent variable (HR: 12.19, *p* < 0.001, Supplementary Table S6). 

### 3.6. Validation of the Results

In univariate analysis of the TCGA cohort TB in 1 HPF and the MELF pattern of invasion yielded prognostic significance in the subgroup of MSI tumors. In contrast to TB, for which the prognostic impact depending on molecular subgroups and the appropriate cutoff has yet to be determined, the MELF pattern has been shown to be prognostic in several previous studies. Therefore, we decided to proceed with a validation of the novel cutoff for TB in two separate cohorts and to forego a validation of MELF.

In the Munich validation cohort, TB in 1 HPF was able to stratify patients regarding DSS (5-year median survival 79.2% vs. 37.5%, *p* = 0.042, Supplementary Figure S3A), OS (5-year median survival 75.2% vs. 20.0%, *p* = 0.002) and PFS (5-year median survival 83.7% vs. 50.0%, *p* = 0.010) in univariate analysis.

In multivariate analysis (including age, FIGO stage, LVSI and TB in 1 HPF), TB in 1 HPF remained as an independent prognostic factor for DSS (HR: 4.93, *p* = 0.032), OS (HR: 4.57, *p* = 0.017) and PFS (HR: 9.45, *p* = 0.026). Results for multivariate analyses of the Munich validation cohort are shown in Supplementary Table S7.

In the CPTAC validation cohort, only log-rank tests were conducted due to the small cohort size with limited events.

Here, TB in 1 HPF showed prognostic significance for OS (median survival 35.4 months vs. 9.6 months, *p* = 0.005, Supplementary Figure S3B) and PFS (median survival n.a. vs. 17.1 months, *p* = 0.008). 

## 4. Discussion

The molecular classification of endometrial cancer provided by the extensive molecular work-up by the TCGA program has provided major advantages for prognostic patient stratification [3]. It could be shown that *POLE* mutated cases are associated with good outcomes, whereas CN-high cases show the worst prognosis [5]. The two subgroups comprising the majority of cases, MSI and CN-low tumors, are associated with intermediate outcomes. This makes treatment planning (e.g., extent of lymphadenectomy, adjuvant treatment strategies) extremely challenging [43].

Comprehensively analyzing histomorphologic characteristics, we aimed to identify major tissue-based prognostic parameters for MSI and CN-low endometrioid endometrial cancer—in particular aiming to characterize easy-to-assess biomarkers for clinical utility in daily practice. In univariate analysis, several parameters yielded prognostic significance in the whole cohort combining MSI and CN-low cases. After stratification of the patients according to TCGA-defined molecular subgroups, multivariate analysis revealed TB in 1 HPF as an independent prognostic factor in the subgroup of MSI tumors, and the prognostic relevance of TB in 1 HPF could be validated for MSI cases in two independent cohorts. These results are in line with previous publications reporting inferior outcomes for tumors with high TB compared to tumors with low TB for several tumor entities like colorectal adenocarcinomas, head and neck squamous cell carcinomas and non-small cell lung cancer [17]. Up to date, there are only a few publications reporting inferior outcomes of patients with high TB in endometrial cancer [19,20]. In general, TB is supposed to be associated with at least partial epithelial to mesenchymal transition (EMT), causing cancer invasion and leading to lymph node metastases and distant metastases [44]. In our study, we confirmed these findings with a positive correlation between the number of tumor buds and the number of lymph node metastases and an association between high TB and pN+ status. Hence, considering the results for TB in MSI cases might help in selecting patients who might benefit from lymphadenectomy [45].

From a clinical point of view, we believe that the evaluation of TB should routinely be included in pathology reports as it could complement established risk assessment for several reasons: (1) So far, mainly grading, depth of myometrial invasion and LVSI are utilized as pathologic parameters for the prognostic stratification of patients [8,46,47]. As TB was shown to be an independent prognostic factor, it allows for an even more precise prognostic stratification of patients, especially of patients with prognostically unclear MSI/CN-low tumors. (2) TB is an easily applicable characteristic, which can be assessed time and cost effectively on H&E-stained slides [19]. (3) We and others could demonstrate a high interobserver concordance for the determination of TB in previous publications, e.g., in squamous cell carcinomas of the head and neck and the lung, but also in endometrial adenocarcinomas [20,48].

Interestingly, we could observe a higher rate of high TB in MSI tumors compared to CN-low tumors. For colorectal cancer, TB is mainly detected in tumors with proficient mismatch repair status [49]. It is hypothesized that the higher number of immune cells in tumors with MSI might disrupt any buds in these cases [50]. Hence, further studies should be undertaken analyzing the composition (and maybe modulation) of the immune microenvironment in the context of TB in endometrioid adenocarcinomas.

While we included various methodologies for determining tumor budding (TB in 1 HPF, TB in 10 HPF, ITBCC scheme) and obtained significant results from the different methodologies in univariate analysis, TB in 1 HPF outperformed the other methodologies in multivariate analysis. Thus, we propose the evaluation of TB in 1 HPF for implementation in clinical practice. It has to be kept in mind that there is one main constraint when comparing our results for TB with other publications—with our results adding valuable novel findings to the up to date available data: While we recorded absolute values for the number of tumor buds, which allowed us to conduct a systematic cutoff identification (utilizing “Cutoff Finder” [40]) and to identify prognostically optimal cutoffs for patient stratification (a two-tiered scheme with TB-low: 0–2 buds and TB-high ≥ 3 buds), other groups conducted different approaches. Qi et al. applied a cutoff of five buds/HPF after receiver operating characteristic analysis, whereas Rau et al. and Yamamoto et al. stratified into budding absent vs. budding present [19,20,51]. For colorectal cancer, a recent ITBCC consensus paper was published reporting the evaluation criteria and cutoffs to apply [41]. However, for other tumor entities like endometrial cancer, a comparable consensus is, to date, lacking. An effort to reach such a consensus for endometrial cancer should be the aim of future publications, especially as the utilization of different thresholds for TB evaluation might hinder the acceptance of TB in clinical routine. 

By contrast, in the subgroup of CN-low tumors, none of the evaluated histomorphologic parameters (TB, MCNS, MELF, TSR (describing the severity of stromal desmoplasia), TIL) could add prognostic information to the established parameters like age, FIGO stage and LVSI. The probed morphologic patterns failed to reach prognostic significance, while the “old-fashioned” established grading as defined by the WHO classification remained as the sole tissue-based independent prognostic biomarker [34]. This finding underlines the major value of histomorphology and histomorphologic grading based on gland formation and nuclear pleomorphism for patient prognostication. In an era of molecular biology, WHO-based grading can help to guide patient management and therapeutic decisions in women with CN-low endometrial cancers with intermediate risk [46]. Interestingly, Rau et al. could demonstrate inferior outcomes of patients with TB in the CN-low subgroup [19]. These inconclusive results warrant further studies, ideally on larger cohorts to clarify the implications of TB in CN-low tumors.

As discussed, complexity of histopathological patterns and their prognostic relevance are influenced by molecular subgroups defined by TCGA. As recently shown by Adamczyk-Gruszka et al., not only molecular subgrouping but also distinct mutations might influence the cancer cell–stroma interaction, and therewith the morphologic features. In their recent publication, the impact of *FGFR2* mutations on EMT (and TB as one of the EMT hallmarks) was described [52]. Furthermore, e.g., aberrant ß-catenin expression was strongly correlated with TB (reviewed by [18]). Future studies are required to understand the complex relationships between mutational patterns and histomorphologic appearance of EC. 

Confirming previous studies, we observed a significant correlation of high-grade TB with high-grade histomorphology as defined by WHO grading [19,53]. Both morphologic patterns, high WHO grade and TB represent distinct features of aggressiveness and invasive potential. They might be the morphologic manifestation of a gradual mechanism (maybe driven by underlying molecular events as the above-discussed *FGFR2* mutation) by which an adenocarcinoma loses the ability to form glands, leading to formation of solid areas from which invasive cell clusters in the sense of TB can detach. The close correlation of both features shows that they are two distinct histomorphologic manifestations of an aggressive biologic potential and unfavorable patient prognosis. Both biomarkers should be reported by pathologists as important tissue-based biomarkers.

Almangush et al. could demonstrate a high concordance between the results obtained for TB in biopsy specimens and resection specimens [54]. Therefore, a further interesting clinical aspect would be the applicability of TB evaluation on endometrial curettage specimens for the improvement of treatment planning. At least, in our study, the most significant results for TB were determined by the analysis of 1 HPF in resection specimens, rendering the approach applicable to biopsies and curettage specimens. As we could not directly utilize our approach on endometrial curettage specimens, additional studies, which analyze the applicability of TB assessment in endometrial curettage specimens and their implications on clinical care are required.

Our study has some limitations. For the TCGA cohort, only one digitized slide per case was available. Thus, on the one hand, cases had to be excluded where no appropriate scanned slide was available (diminishing the potential cohort size), and on the other hand, no additional studies (e.g., immunohistochemical analyses of the composition of the immune infiltrate) could be conducted. Furthermore, no data on treatment procedures could be evaluated, limiting the transferability of our results on therapy response prediction.

In addition, while we could validate the prognostic significance of TB in three independent cohorts, the sizes of the validation cohorts were relatively small. Therefore, the prognostic significance and the cutoffs applied in our study should be validated in future studies on larger cohorts. The RAINBO umbrella trial (refining adjuvant treatment in endometrial cancer based on molecular features) is currently recruiting and may provide interesting cohorts for further analyses (https://clinicaltrials.gov/ct2/show/NCT05255653 (accessed on 9 January 2022)).

## 5. Conclusions

TB was shown to be a promising histomorphologic parameter in the subgroup of MSI tumors, while WHO-defined grading was found to be the only independent tissue-based biomarker in CN-low carcinomas. Hence, morphological grading and the evaluation of TB might help to identify high-risk patients who could benefit from postsurgical adjuvant treatment or low-risk groups in whom omission of lymphadenectomy might be an option. Further studies are required to standardize the methodology of TB assessment, to evaluate the applicability of TB determination in biopsy specimens and to investigate the predictive relevance of TB. For CN-low tumors, none of the analyzed histomorphologic parameters could add prognostic significance to the established clinical and pathological risk factors.

## Figures and Tables

**Table 1:** Clinicopathologic characteristics of the TCGA study cohort as well as the Munich and CPTAC validation cohorts.

	TCGA		Munich		CPTAC	
	Number of Patients with Available Data	%	Number of Patients with Available Data	%	Number of Patients with Available Data	%
Age (median, interquartile range)	61.0, 13.0		69.7, 11.8		64.2, 5.7	
N/A	2		0		0	
FIGO stage						
I	172	75.4	28	59.6	21	84.0
II	19	8.3	9	19.1	2	8.0
III	34	14.9	6	12.8	2	8.0
IV	3	1.3	4	8.5	0	0
Grading						
G1	61	26.8	9	19.1	8	32.0
G2	106	46.5	14	29.8	13	52.0
G3	61	26.8	24	51.1	4	16.0
pT						
1	120	75.9	29	61.7	20	83.3
2	23	14.6	11	23.4	2	8.3
3	15	9.5	7	14.9	2	8.3
4	0	0	0	0	0	0
N/A	70		0	0	1	
pN						
N0	187	85.0	43	91.5	16	66.7
N1/N2	21	9.5	4	8.5	0	0
NX	12	5.5	0	0	8	33.3
N/A	8		0	0	1	
Lymphovascular space invasion						
absent	187	82.0	33	75.0	17	94.4
present	41	18.0	11	25.0	1	5.6
N/A	0		3		7	
Perineural invasion						
absent	226	99.1	42	95.5	18	100
present	2	0.9	2	4.5	0	
N/A	0		0		7	
Residual tumor						
R0	163	88.1	41	89.1	14	60.9
R1/R2	10	5.0	3	6.5	2	8.6
RX	12	6.5	2	4.3	7	30.4
N/A	43		1		2	
Subtype						
MSI	107	46.9	44	100	25	100
CN-LOW	121	53.1	-	-	-	-

**Table 2:** Clinicopathologic associations in the TCGA study cohort.

	Grading			TB in 1 HPF			TB in 10 HPF			Minimal Cell Nest Size			TSR			TIL			MELF		
	Low	High	*p*	Low	High	*p*	Low	High	*p*	Small	Big	*p*	Low	High	*p*	Low	High	*p*	Absent	Present	*p*
Age (median; IQR)	60 (12.5)	62 (17)	0.632	61 (12.5)	60 (14.5)	0.446	60.5 (13)	64.5 (19.5)	0.899	59.5 (14.5)	61 (12.25)	0.308	61.5 (17.25)	61 (13)	0.844	61 (13)	60.5 (10.75)	0.974	61 (15)	61 (7.5)	0.919
N/A	2																		2		
pT																					
1	90 (80.4)	30 (65.2)	0.108	110 (76.4)	10 (71.4)	0.259	114 (78.1)	6 (50.0)	**0.045**	17 (70.8)	103 (76.9)	0.401	5 (50.0)	115 (77.7)	**0.012**	115 (76.2)	5 (71.4)	0.194	103 (76.9)	30 (69.8)	0.227
2	14 (12.5)	9 (19.6)		22 (15.3)	1 (7.1)		20 (13.7)	3 (25.0)		3 (12.5)	20 (14.9)		1 (10.0)	22 (14.9)		23 (15.2)	0 (0.0)		20 (14.9)	5 (11.6)	
3	8 (7.1)	7 (15.2)		12 (8.3)	3 (21.4)		12 (8.2)	3 (25.0)		4 (16.7)	11 (8.2)		4 (40.0)	11 (7.4)		13 (8.6)	2 (28.6)		11 (8.2)	8 (18.6)	
N/A	70																		70		
pN																					
0	142 (88.8)	45 (75.0)	**0.001**	176 (87.6)	11 (57.9)	**<0.001**	177 (87.2)	10 (58.8)	**0.007**	23 (67.6)	164 (88.2)	**0.003**	12 (75.0)	175 (85.8)	0.249	179 (85.2)	8 (80.0)	0.324	164 (86.3)	41 (77.4)	**0.007**
+	8 (5.0)	13 (21.7)		14 (7.0)	7 (36.8)		16 (7.9)	5 (29.4)		9 (26.5)	12 (6.5)		3 (18.8)	18 (8.8)		19 (9.0)	2 (20.0)		14 (7.4)	11 (20.8)	
X	10 (6.2)	2 (3.3)		11 (5.5)	1 (5.3)		10 (4.9)	2 (11.8)		2 (5.9)	10 (5.4)		1 (6.2)	11 (5.4)		12 (5.7)	0 (0.0)		12 (6.3)	1 (1.9)	
N/A	8																		8		
FIGO stage																					
1/2	146 (87.4)	45 (73.8)	**0.024**	178 (85.6)	13 (65.0)	**0.026**	178 (84.8)	13 (72.2)	0.182	27 (75.0)	164 (85.4)	0.139	10 (62.5)	181 (85.4)	**0.028**	185 (84.9)	6 (60.0)	0.060	169 (85.8)	39 (72.2)	**0.028**
3/4	21 (12.6)	16 (26.2)		30 (14.4)	7 (35.0)		32 (15.2)	5 (27.8)		9 (25.0)	28 (14.6)		6 (37.5)	31 (14.6)		33 (15.1)	4 (40.0)		28 (14.2)	15 (27.8)	
Grading																					
Low-grade	-	-	-	165 (79.3)	2 (10.0)	**<0.001**	164 (78.1)	3 (16.7)	**<0.001**	9 (25.0)	158 (82.3)	**<0.001**	11 (68.8)	156 (73.6)	0.770	162 (74.3)	5 (50.0)	0.137	144 (73.1)	37 (68.5)	>0.999
High-grade	-	-		43 (20.7)	18 (90.0)		46 (21.9)	15 (83.3)		27 (75.0)	34 (17.7)		5 (31.2)	56 (26.4)		56 (25.7)	5 (50.0)		53 (26.9)	17 (31.5)	
Lymphovascular space invasion																					
Absent	146 (87.4)	41 (67.2)	**<0.001**	174 (83.7)	13 (65.0)	0.061	177 (84.3)	10 (55.6)	**0.006**	25 (69.4)	162 (84.4)	0.055	11 (68.8)	176 (83.0)	0.175	178 (81.7)	9 (90.0)	0.695	164 (83.2)	44 (81.5)	0.322
Present	21 (12.6)	20 (32.8)		34 (16.3)	7 (35.0)		33 (15.7)	8 (44.4)		11 (30.6)	30 (15.6)		5 (31.2)	36 (17.0)		40 (18.3)	1 (10.0)		33 (16.8)	10 (18.5)	
Perineural invasion																					
Absent	167 (100)	59 (96.7)	0.071	207 (99.5)	19 (95.0)	0.168	209 (99.5)	17 (94.4)	0.152	35 (97.2)	191 (99.5)	0.291	15 (93.8)	211 (99.5)	0.136	216 (99.1)	10 (100.0)	> 0.999	195 (99.0)	54 (100.0)	>0.999
Present	0 (0.0)	2 (3.3)		1 (0.5)	1 (5.0)		1 (0.5)	1 (5.6)		1 (2.8)	1 (0.5)		1 (6.2)	1 (0.5)		2 (0.9)	0 (0.0)		2 (1.0)	0 (0.0)	
Residual tumor																					
R0	125 (91.9)	38 (77.6)	**<0.001**	152 (90.5)	11 (64.7)	**<0.001**	154 (90.6)	9 (60.0)	**0.002**	21 (72.4)	142 (91.0)	**0.002**	13 (92.9)	150 (87.7)	0.511	156 (89.1)	7 (70.0)	0.101	144 (90.0)	38 (84.4)	**0.048**
R1/R2	2 (1.5)	8 (16.3)		5 (3.0)	5 (29.4)		6 (3.5)	4 (26.7)		6 (20.7)	4 (2.6)		1 (7.1)	9 (5.3)		9 (5.1)	1 (10.0)		6 (3.8)	5 (11.1)	
RX	9 (6.6)	3 (6.1)		11 (6.5)	1 (5.9)		10 (5.9)	2 (13.3)		2 (6.9)	10 (6.4)		0 (0.0)	12 (7.0)		10 (5.7)	2 (20.0)		10 (6.2)	2 (4.4)	
N/A	43																		43		
Subtype																					
MSI	63 (37.7)	44 (72.1)	**<0.001**	93 (44.7)	14 (70.0)	**0.036**	93 (44.3)	14 (77.8)	**0.007**	25 (69.4)	82 (42.7)	**0.004**	7 (43.8)	100 (47.2)	>0.999	99 (45.4)	8 (80.0)	**0.049**	92 (46.7)	28 (51.9)	0.846
CN-LOW	104 (62.3)	17 (27.9)		115 (55.3)	6 (30.0)		117 (55.7)	4 (22.2)		11 (30.6)	110 (57.3)		9 (56.2)	112 (52.8)		119 (54.6)	2 (20.0)		105 (53.3)	26 (48.1)	
Depth of myometrial invasion																					
Inner half	113 (75.3)	19 (40.4)	**<0.001**	123 (68.0)	9 (56.2)	0.407	127 (69.0)	5 (38.5)	**0.033**	14 (46.7)	118 (70.7)	**0.019**	8 (57.1)	124 (67.8)	0.556	128 (67.4)	4 (57.1)	0.686	120 (69.4)	23 (51.1)	0.056
Outer half	37 (24.7)	28 (59.6)		58 (32.0)	7 (43.8)		57 (31.0)	8 (61.5)		16 (53.3)	49 (29.3)		6 (42.9)	59 (32.2)		62 (32.6)	3 (42.9)		53 (30.6)	22 (48.9)	
N/A	31																				

**Table 3:** Results for the log-rank tests for disease-specific (DSS), overall survival (OS) and progression-free survival (PFS) for the whole TCGA cohort as well as for the MSI and CN-LOW subgroups.

		Grading (Low vs. High)	TB in 1 HPF (Low vs. High)	TB in 10 HPF (Low vs. High)	MCNS (Low vs. High)	TSR (Low vs. High)	TIL (Low vs. High)	MELF (Absent vs. Present)	Lymphovascular Space Invasion (Absent vs. Present)	ITBCC Grading Scheme (Bd1 vs. Bd2 vs. Bd3)
**TCGA**	**DSS**	**<0.001**	**<0.001**	**<0.001**	**<0.001**	**0.022**	0.276	**0.028**	**<0.001**	**<0.001**
	**OS**	**<0.001**	**<0.001**	**<0.001**	**<0.001**	**0.014**	0.077	0.549	0.078	**<0.001**
	**PFS**	**0.011**	**<0.001**	**0.001**	0.066	0.879	0.813	0.987	**0.034**	**0.026**
**MSI**	**DSS**	**0.007**	**<0.001**	**<0.001**	**0.002**	**0.004**	0.503	**0.014**	**0.008**	**0.001**
	**OS**	0.067	**<0.001**	**<0.001**	**<0.001**	**0.034**	0.403	0.217	0.115	**<0.001**
	**PFS**	**0.045**	**<0.001**	**0.007**	0.066	0.334	0.230	0.258	0.339	0.064
**CN-low**	**DSS**	**<0.001**	0.061	**0.043**	0.212	0.735	**<0.001**	0.666	**0.041**	0.168
	**OS**	**0.010**	0.377	0.326	0.723	0.215	**<0.001**	0.644	0.523	0.682
	**PFS**	0.240	0.177	0.115	0.692	0.366	**0.034**	0.281	**0.049**	0.483

**Table 4:** Results of multivariate analysis for disease-specific survival for those parameters that showed significant *p*-values in univariate analysis.

	TB in 1 HPF			TB in 10 HPF			Minimal Cell Nest Size		
		HR	*p*		HR	*p*		HR	*p*
TCGA	Age	0.95 (0.90–1.01)	0.091	Age	0.95 (0.89–1.01)	0.085	Age	0.94 (0.88–1.00)	0.061
	FIGO stage 3/4 (1/2 = 1)	2.72 (0.70–10.52)	0.148	FIGO stage 3/4 (1/2 = 1)	3.14 (0.83–11.91)	0.093	FIGO stage 3/4 (1/2 = 1)	3.76 (1.06–13.36)	**0.040**
	Grading high-grade (low-grade = 1)	7.60 (1.47–39.22)	**0.015**	Grading high-grade (low-grade = 1)	9.03 (1.81–45.17)	**0.007**	Grading high-grade (low-grade = 1)	9.89 (1.85–52.91)	**0.007**
	LVSI present (absent = 1)	5.83 (1.57–21.66)	**0.008**	LVSI present (absent = 1)	5.22 (1.43–19.00)	**0.012**	LVSI present (absent = 1)	5.38 (1.48–19.58)	**0.011**
	TB in 1 HPF high (low = 1)	3.41 (0.76–15.36)	0.111	TB in 10 HPF high (low = 1)	2.21 (0.48–10.31)	0.311	Minimal cell nest size (absent = 1)	1.31 (0.29–5.90)	0.726
MSI	Age	0.96 (0.89–1.04)	0.296	Age	0.97 (0.89–1.05)	0.414	Age	0.98 (0.90–1.06)	0.576
	FIGO stage 3/4 (1/2 = 1)	7.33 (1.19–45.08)	**0.032**	FIGO stage 3/4 (1/2 = 1)	6.75 (1.16–39.14)	**0.033**	FIGO stage 3/4 (1/2 = 1)	7.60 (1.40–41.25)	**0.019**
	Grading high-grade (low-grade = 1)	4.08 (0.36–46.58)	0.257	Grading high grade (low-grade = 1)	5.66 (0.57–55.89)	0.138	Grading high-grade (low-grade = 1)	4.46 (0.40–49.44)	0.223
	LVSI present (absent = 1)	1.79 (0.31–10.38)	0.518	LVSI present (absent = 1)	1.62 (0.27–9.80)	0.598	LVSI present (absent = 1)	2.68 (0.56–12.87)	0.219
	TB in 1 HPF high (low = 1)	11.90 (1.53–92.36)	**0.018**	TB in 10 HPF high (low = 1)	6.48 (0.89–46.94)	0.064	Minimal cell nest size (absent = 1)	3.72 (0.58–24.04)	0.167
CN-low	Age	0.81 (0.67–0.99)	**0.039**	Age	0.81 (0.67–0.99)	**0.039**	Age	0.82 (0.67–0.99)	**0.036**
	FIGO stage 3/4 (1/2 = 1)	0.31 (0.00–73.02)	0.676	FIGO stage 3/4 (1/2 = 1)	0.31 (0.00–68.35)	0.669	FIGO stage 3/4 (1/2 = 1)	0.91 (0.00–1437.06)	0.979
	Grading high-grade (low-grade = 1)	37.78 (1.88–757.32)	**0.018**	Grading high-grade (low-grade = 1)	37.73 (1.87–760.53)	**0.018**	Grading high-grade (low-grade = 1)	41.91 (2.29–767.91)	**0.012**
	LVSI present (absent = 1)	24.61 (1.19–509.08)	**0.038**	LVSI present (absent = 1)	24.67 (1.20–508.78)	**0.038**	LVSI present (absent = 1)	20.76 (0.91–471.82)	0.057
	TB in 1 HPF high (low = 1)	0.72 (0.00–181.61)	0.907	TB in 10 HPF high (low = 1)	0.74 (0.00–179.96)	0.913	Minimal cell nest size (absent = 1)	0.21 (0.00–579.03)	0.703
	TSR			MELF			ITBCC scheme		
		HR	*p*		HR	*p*		HR	*p*
TCGA	Age	0.93 (0.87–0.99)	**0.017**	Age	0.97 (0.90–1.04)	0.341	Age	0.95 (0.89–1.01)	0.089
	FIGO stage 3/4 (1/2 = 1)	2.95 (0.77–11.30)	0.115	FIGO stage 3/4 (1/2 = 1)	2.64 (0.66–10.53)	0.169	FIGO stage 3/4 (1/2 = 1)	3.08 (0.79–11.95)	0.105
	Grading high-grade (low-grade = 1)	11.61 (2.43–55.53)	**0.002**	Grading high-grade (low-grade = 1)	9.29 (1.90–45.42)	**0.006**	Grading high-grade (low-grade = 1)	8.31 (1.57–44.09)	**0.013**
	LVSI present (absent = 1)	4.25 (1.11–16.21)	**0.034**	LVSI present (absent = 1)	4.94 (1.22–19.98)	**0.025**	LVSI present (absent = 1)	6.22 (1.61–24.00)	**0.008**
	TSR (high = 1)	4.04 (0.59–27.82)	0.156	MELF (absent = 1)	2.81 (0.68–11.54)	0.152	ITBCC scheme (Bd1/Bd2 = 1)	2.21 (0.42–11.61)	0.349
MSI	Age	0.94 (0.86–1.02)	0.133	Age	1.03 (0.94–1.14)	0.521	Age	0.98 (0.90–1.06)	0.575
	FIGO stage 3/4 (1/2 = 1)	5.19 (0.87–30.94)	0.070	FIGO stage 3/4 (1/2 = 1)	8.29 (0.97–70.51)	0.053	FIGO stage 3/4 (1/2 = 1)	6.05 (1.08–33.98)	**0.041**
	Grading high-grade (low-grade = 1)	9.29 (1.06–81.38)	**0.044**	Grading high-grade (low-grade = 1)	6.09 (0.45–82.29)	0.174	Grading high-grade (low-grade = 1)	3.97 (0.36–44.35)	0.263
	LVSI present (absent = 1)	1.79 (0.30–10.67)	0.524	LVSI present (absent = 1)	5.21 (0.57–47.97)	0.145	LVSI present (absent = 1)	3.27 (0.64–16.74)	0.155
	TSR (high = 1)	5.38 (0.51–56.31)	0.160	MELF (absent = 1)	14.14 (1.52–131.48)	**0.020**	ITBCC scheme (Bd1/Bd2 = 1)	5.00 (0.72–34.68)	0.103
CN-low	Age	0.81 (0.67–0.99)	**0.038**	Age	0.82 (0.68–0.99)	**0.039**	Age	0.81 (0.67–0.99)	**0.035**
	FIGO stage 3/4 (1/2 = 1)	0.24 (0.01–10.75)	0.463	FIGO stage 3/4 (1/2 = 1)	0.27 (0.00–201.15)	0.701	FIGO stage 3/4 (1/2 = 1)	0.76 (0.00–2271.29)	0.947
	Grading high-grade (low-grade = 1)	35.18 (1.95–636.22)	**0.016**	Grading high-grade (low-grade = 1)	30.37 (1.68–548.13)	**0.021**	Grading high-grade (low-grade = 1)	40.88 (2.15–778.76)	**0.014**
	LVSI present (absent = 1)	24.55 (1.22–492.96)	**0.036**	LVSI present (absent = 1)	24.82 (1.24–496.83)	**0.036**	LVSI present (absent = 1)	22.47 (1.04–484.32)	**0.047**
	TSR (high = 1)	0.00 (0.00–Inf)	0.999	MELF (absent = 1)	0.94 (0.00–417.53)	0.984	ITBCC scheme (Bd1/Bd2 = 1)	0.27 (0.00–1388.68)	0.762

## Data Availability

Datasets of our analyses can be requested from the corresponding author.

## Supplementary Materials

The following supporting information can be downloaded at: https://www.mdpi.com/article/10.3390/cancers15153832/s1: Figure S1: Spearman correlations between histomorphological parameters analyzed (HPF: high-power field, TSR: tumor–stroma ratio). Figure S2: Univariate survival analysis for disease-specific survival (whole TCGA cohort). Figure S3: Kaplan—Meier plots for tumor budding in 1 HPF for disease-specific survival (Munich validation cohort) (A) and for tumor budding in 1 HPF for overall survival (CPTAC validation cohort) (B). Table S1: Metrics of the histomorphological parameters obtained in the TCGA study cohort. Table S2: Cutoffs identified with “Cutoff Finder” [40] and dispersion of patients of the TCGA cohort according to the identified cutoffs. Table S3: Correlations between each histomorphological parameter were calculated with a Fisher’s exact test and resulting *p*-values are shown. Table S4: Results for the Log-rank tests for disease-specific (DSS), overall survival (OS) and progression-free survival (PFS) for the whole TCGA cohort as well as for the MSI and CN-LOW subgroups. Table S5: Five-year survival rates are shown for disease-specific (DSS), overall survival (OS) and progression-free survival (PFS) for the whole TCGA cohort as well as for the MSI and CN-LOW subgroups. Table S6: Multivariate analysis including tumor budding and MELF pattern of invasion (disease-specific survival). Table S7: Results of multivariate analysis for the Munich validation cohort.

## Abbreviations

CNV	Copy number variation
CPTAC	Clinical Proteomic Tumor Analysis Consortium
DSS	Disease-specific survival
FIGO	Fédération Internationale de Gynécologie et d’Obstétrique
GDC	Genomic Data Commons
H&E	Hematoxylin and eosin
HPF	High-power field
LVSI	Lymphovascular space invasion
MELF	Microcystic, elongated, fragmented
MSI	Microsatellite instability
OS	Overall survival
PFS	Progression-free survival
POLE	Polymerase ε
TB	Tumor budding
TCGA	The Cancer Genome Atlas
TIL	Tumor infiltrating lymphocytes
TSR	Tumor–stroma ratio/stromal desmoplasia
UCEC	Uterine Corpus Endometrial Carcinoma
WHO	World Health Organization
