# Supplementary material for: Independent Tissue-Based Biomarkers in Endometrioid Endometrial Cancer: Tumor Budding in Microsatellite Instability and WHO Grading in Copy-Number-Low Patients
Source: Cancers (Basel). 2023 Jul 28;15(15):3832. doi: 10.3390/cancers15153832 (PMC10416921; doi:10.3390/cancers15153832)
Supplement: Supplementary file 1 [file cancers-15-03832-s001.zip › cancers-2452557-supplementary.pdf]

Supplementary tables

Supplementary Table S1: Metrics of the histomorphological parameters obtained in the TCGA study cohort.

	median	interquartile range	mean	standard deviation	min	max
TB in 1 HPF	0.0	1.0	0.7	1.3	0.0	9.0
TB in 10 HPF	0.0	1.0	1.3	2.9	0.0	20.0
Minimal cell nest size	8.0	13.3	13.0	15.0	1.0	95.0
Tumor-stroma ratio	3.5	3.3	4.1	2.5	0.4	19.0
Inflammation	8.0	11.0	10.9	9.7	1.0	50.0

Supplementary Table S2: Cutoffs identified with “Cutoff Finder” [40] and dispersion of patients of the TCGA cohort according to the identified cutoffs.

	Grading		TB in 1 HPF		TB in 10 HPF		Minimal cell nest size		TSR		Inflammation		MELF		Lymphovascular space invasion	
	low grade	high grade	low	high	low	high	small	big	low	high	low	high	absent	present	absent	present
Cutoff	G1, G2	G3	0-2	≥ 3	0-5	≥ 6	1	≥ 2	0-1.38	≥ 1.38	0-32	≥ 33	-	-	-	-
n	167	61	208	20	210	18	36	192	16	212	218	10	174	31	187	41
%	73.2	26.8	91.2	8.8	92.1	7.9	15.8	84.2	7.0	93.0	95.6	4.4	84.9	15.1	82.0	18.0

Supplementary Table S3: Correlations between each histomorphological parameter were calculated with a Fisher's exact test and resulting p-values are shown.

	TB in 1 HPF (low vs. high)	TB in 10 HPF (low vs. high)	MCNS (low vs. high)	TSR (low vs. high)	Inflammation (low vs. high)	MELF (absent vs. present)	Lymphovascular space invasion (absent vs. present)	ITBCC grading scheme (Bd1 vs. Bd2 vs. Bd3)
TB in 1 HPF (low vs. high)		**<0.001**	**<0.001**	0.638	> 0.999	0.499	0.061	**<0.001**
TB in 10 HPF (low vs. high)			**<0.001**	> 0.999	0.568	0.766	**0.006**	**<0.001**
MCNS (low vs. high)				0.723	> 0.999	0.282	0.055	**<0.001**
TSR (low vs. high)					0.149	> 0.999	0.175	0.926
Inflammation (low vs. high)						0.628	0.695	0.714
MELF (absent vs. present)							0.322	**0.004**
Lymphovascular space invasion (absent vs. present)								**0.008**
ITBCC grading scheme (Bd1 vs. Bd2 vs. Bd3)								

Supplementary Table S4: Results for the Log-rank tests for disease-specific (DSS), overall survival (OS) and progression-free survival (PFS) for the whole TCGA cohort as well as for the MSI and CN-LOW subgroups.

		**Grading (low vs. high)**	**TB in 1 HPF (low vs. high)**	**TB in 10 HPF (low vs. high)**	**MCNS (low vs. high)**	**TSR (low vs. high)**	**Inflammation (low vs. high)**	**MELF (absent vs. present)**	**Lymphovascular space invasion (absent vs. present)**	**ITBCC grading scheme (Bd1 vs. Bd2 vs. Bd3)**
**TCGA**	**DSS**	**<0.001**	**<0.001**	**<0.001**	**<0.001**	**0.022**	0.276	**0.028**	**<0.001**	**<0.001**
	**OS**	**<0.001**	**<0.001**	**<0.001**	**<0.001**	**0.014**	0.077	0.549	0.078	**<0.001**
	**PFS**	**0.011**	**<0.001**	**0.001**	0.066	0.879	0.813	0.987	**0.034**	**0.026**
**MSI**	**DSS**	**0.007**	**<0.001**	**<0.001**	**0.002**	**0.004**	0.503	**0.014**	**0.008**	**0.001**
	**OS**	0.067	**<0.001**	**<0.001**	**<0.001**	**0.034**	0.403	0.217	0.115	**<0.001**
	**PFS**	**0.045**	**<0.001**	**0.007**	0.066	0.334	0.230	0.258	0.339	0.064
**CN-low**	**DSS**	**<0.001**	0.061	**0.043**	0.212	0.735	**<0.001**	0.666	**0.041**	0.168
	**OS**	**0.010**	0.377	0.326	0.723	0.215	**<0.001**	0.644	0.523	0.682
	**PFS**	0.240	0.177	0.115	0.692	0.366	**0.034**	0.281	**0.049**	0.483

Supplementary Table S5: Five-year survival rates are shown for disease-specific (DSS), overall survival (OS) and progression-free survival (PFS) for the whole TCGA cohort as well as for the MSI and CN-LOW subgroups.

		Grading		TB in 1 HPF		TB in 10 HPF		MCNS		TSR		Inflammation		MELF		Lymphovascular space invasion		ITBCC grading scheme		
		low	high	low	high	low	high	low	high	low	high	low	high	low	high	absent	present	Bd1	Bd2	Bd3
**TCGA**	**DSS**	97.5	76.5	95.4	35.3	94.9	36.2	67.2	95.2	53.3	93.7	93.3	50.0	95.3	81.6	95.0	78.4	95.8	93.8	52.8
	**OS**	92.7	72.8	91.1	30.3	91.2	27.1	60.0	91.2	49.5	89.3	89.0	45.0	89.2	81.6	89.5	78.4	91.4	91.8	45.2
	**PFS**	84.4	65.0	82.8	27.3	82.0	29.3	59.4	81.7	65.6	79.8	79.4	75.0	81.2	80.9	82.2	66.1	84.2	74.8	45.1
**MSI**	**DSS**	96.7	80.3	94.4	53.5	93.2	51.4	73.5	93.9	0.0	93.4	89.4	100.0	95.3	74.5	94.4	71.1	96.6	89.6	68.7
	**OS**	90.5	76.3	90.3	35.6	90.4	21.4	59.0	90.8	0.0	87.8	84.0	100.0	88.1	74.5	87.9	71.1	92.0	89.6	51.3
	**PFS**	84.8	64.1	81.6	44.3	79.7	51.6	67.3	79.9	0.0	78.8	75.4	100.0	82.1	67.7	79.8	65.1	82.8	74.0	63.7
**CN-LOW**	**DSS**	98.0	72.2	96.2	50.0	96.3	50.0	66.7	96.1	100.0	94.0	96.4	0.0	95.4	87.5	95.4	87.1	95.4	100.0	50.0
	**OS**	93.9	67.7	91.7	50.0	91.8	50.0	66.7	91.4	87.5	90.5	92.8	0.0	89.9	87.5	90.5	87.1	91.1	95.5	50.0
	**PFS**	83.6	66.5	83.4	37.5	83.5	37.5	58.3	82.6	100.0	80.4	82.1	0.0	80.1	91.7	83.5	66.5	84.8	76.0	40.0

Supplementary Table S6: Multivariate analysis including tumor budding and MELF pattern of invasion (disease-specific survival).

	HR	p
TB in 1 HPF high (low=1)	12.19 (3.17 – 46.84)	<0.001
MELF present (absent=1)	3.12 (0.82 – 11.84)	0.095

Supplementary Table S7: Results of multivariate analysis for the Munich validation cohort.

		HR	p
DSS	Age	1.02 (0.96 - 1.07)	0.580
	FIGO stage 3/4 (1/2=1)	2.07 (1.09 - 3.92)	**0.026**
	Grading high grade (low grade=1)	0.43 (0.11 - 1.75)	0.240
	LVSI present (absent=1)	0.63 (0.15 - 2.65)	0.530
	TB in 1 HPF high (low=1)	4.93 (1.15 - 21.2)	**0.032**
OS	Age	1.05 (1.00 - 1.10)	**0.035**
	FIGO stage 3/4 (1/2=1)	1.87 (1.08 - 3.24)	**0.026**
	Grading high grade (low grade=1)	0.62 (0.20 - 1.93)	0.410
	LVSI present (absent=1)	0.66 (0.19 - 2.23)	0.500
	TB in 1 HPF high (low=1)	4.57 (1.31 - 15.98)	**0.017**
PFS	Age	0.96 (0.90 - 1.03)	0.298
	FIGO stage 3/4 (1/2=1)	2.06 (1.00 - 4.25)	0.050
	Grading high grade (low grade=1)	0.80 (0.14 - 4.55)	0.798
	LVSI present (absent=1)	0.42 (0.07 - 2.43)	0.333
	TB in 1 HPF high (low=1)	9.45 (1.31 - 68.18)	**0.026**

Supplementary figures

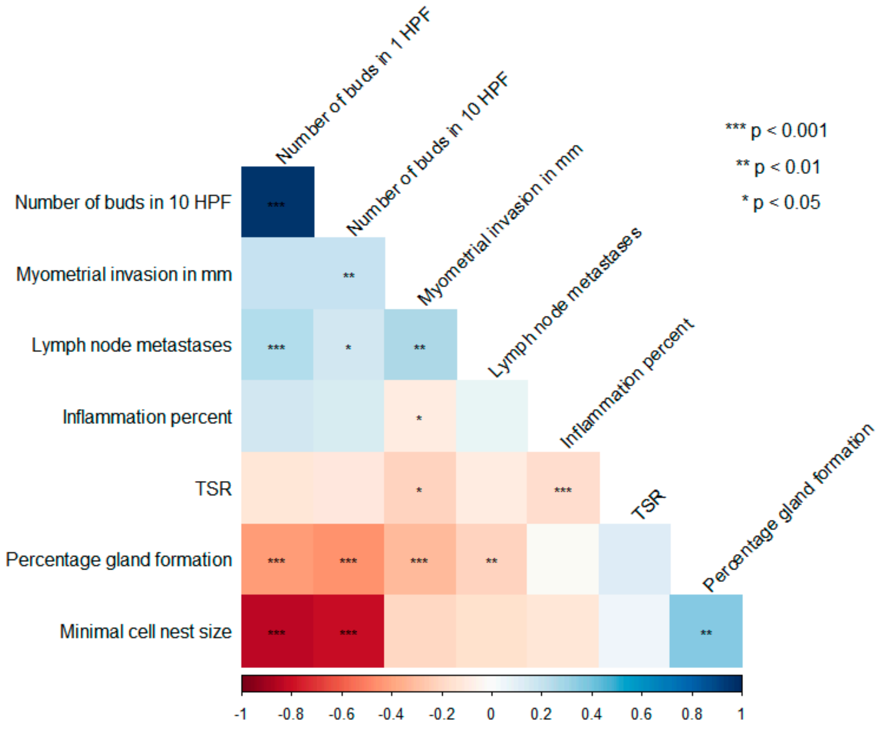

Supplementary Figure S1: Spearman correlations between histomorphological parameters analyzed (HPF: high-power field, TSR: tumor-stroma ratio).

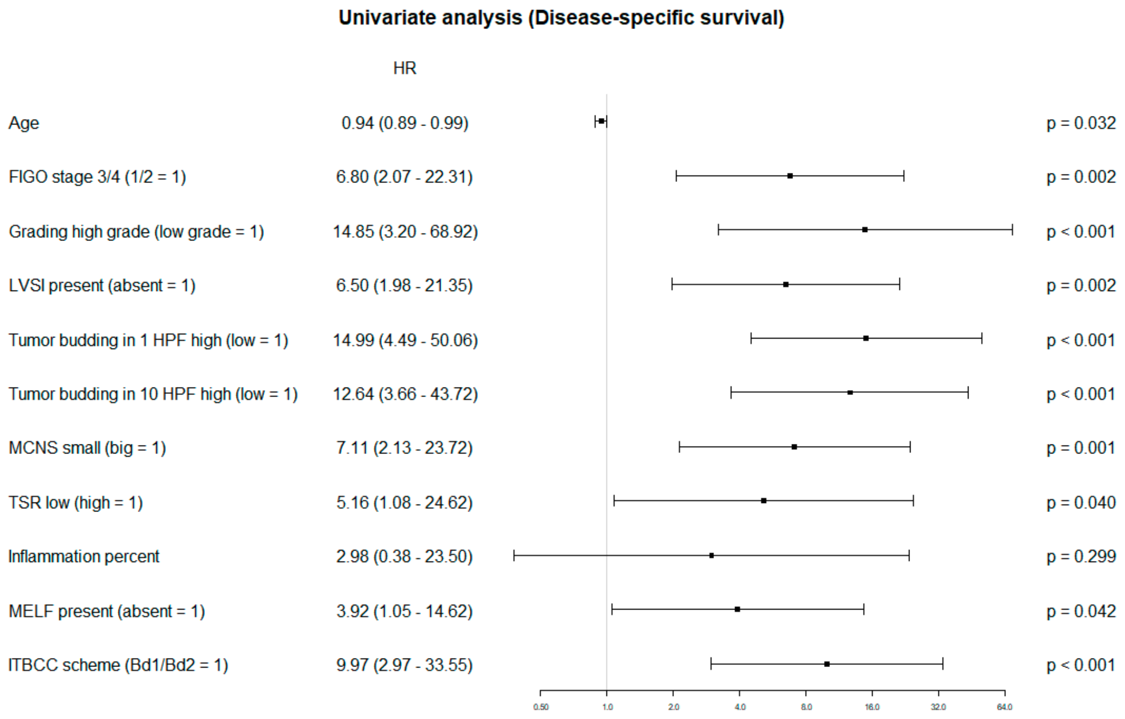

Supplementary Figure S2: Univariate survival analysis for disease-specific survival (whole TCGA cohort).

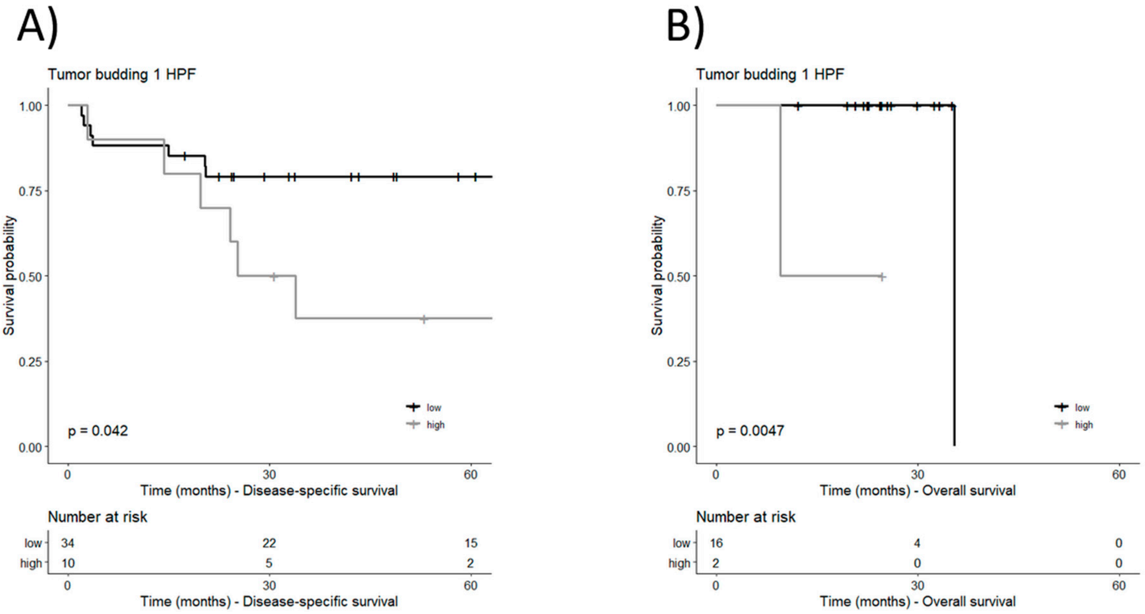

Supplementary Figure S3: Kaplan-Meier plots for tumor budding in 1 HPF for disease-specific survival (Munich validation cohort) (A) and for tumor budding in 1 HPF for overall survival (CPTAC validation cohort) (B).
